# Molecular remodeling of the myocardium in mice with melanocortin-4 receptor deletion before cardiac function impairment

**DOI:** 10.1371/journal.pone.0340465

**Published:** 2026-01-30

**Authors:** Xiaomei Wang, Yuanmin Qi, Ziming Zhu, Caiqin Wang, Zhimin Zhang, Haocheng Jia, Linhui Xia, Kai Meng, Jinxiang Yuan

**Affiliations:** 1 College of Basic Medicine, Jining Medical University, Jining, China; 2 College of Clinical Medicine, Jining Medical University, Jining, China; 3 College of Second Clinical Medicine, Jining Medical University, Jining, China; 4 Lin He’s Academician Workstation of New Medicine and Clinical Translation, Jining Medical University, Jining, China; Brigham and Women's Hospital Division of Cardiovascular Medicine, INDIA

## Abstract

The melanocortin-4 receptor (MC4R) is highly expressed in the hypothalamus, and mutations in this gene are closely associated with the development of hereditary obesity and early-onset severe obesity in humans. *Mc4r* has been shown to be involved in the development of dilated cardiomyopathy. However, the current system for the early diagnosis and treatment of heart disease is not well established. In this study, we analyzed the effects of *Mc4r* knockout on cardiac function, cardiomyocyte morphology, fibrosis, and apoptosis in mice. Moreover, we explored the possible early molecular mechanisms by which *Mc4r* affects cardiac dysfunction via transcriptome sequencing of cardiac cells combined with bioinformatics analysis. Although the overall heart does not show organic changes, our study suggested that cardiomyocytes already show early abnormal changes at the molecular level. The sequencing results revealed that the genes that were differentially expressed between the two groups of mice were enriched mainly in the p53 signaling pathway and the hypoxia-inducible factor 1 (HIF-1) signaling pathway. We screened 10 key target genes via a protein–protein interaction (PPI) network and module analysis. Drugs targeting key genes were subsequently screened, and *angiotensinogen* (*Agt*) and *Kit* were identified as potential drug targets. We analyze relevant data through bioinformatics to screen for signaling pathways and key hub genes that are enriched in differentially expressed genes (DEGs), as well as molecules targeting the hub genes, in order to provide ideas for early prevention of heart disease caused by *Mc4r* gene defects or related obesity.

## 1. Introduction

Heart disease is one of the major causes of increased mortality worldwide [1]. In addition to traditional factors such as poor lifestyle habits, diabetes, and family genetics [2], obesity has become one of the major factors that results in heart disease [3,4]. Currently, common treatments for cardiac disease include cardiac angiography, cardiac catheterization, and surgical stents [5,6]. Although significant progress has been made in treatment, most patients are diagnosed with heart disease after the symptoms manifest. At this stage, the disease is usually irreversible, and treatment is urgently needed but also expensive [7]. Therefore, early preventive and therapeutic detection should be taken as early as possible. Early intervention not only reduces the incidence of heart disease and its tendency to progress to more severe levels but also reduces the suffering and financial burden on patients. Experimental research study is needed to further investigate the molecular mechanisms underlying the early stages of heart disease and provide theoretical guidance for early diagnosis and intervention of heart disease.

Melanocortin-4 receptor (MC4R) is a G protein-coupled transmembrane domain receptor that is highly expressed in the hypothalamus. This molecule plays an important role in the regulation of feeding and energy metabolism [8,9]. Yeo *et al.* [10]first reported an association between *Mc4r* abnormality and weight gain. *Mc4r* deficiency or mutation has emerged as one of the major causes of monogenic obesity in humans [11–13]. Additionally, *Mc4r* is closely associated with the occurrence and progression of multiple diseases, such as cancer, non-alcoholic fatty liver disease, and dilated cardiomyopathy [14–16]. For example, the obesity-related gene *Fto* can increase the risk of breast cancer in conjunction with *Mc4r* [17].

Moreover, increasing evidence suggests an association between *Mc4r* and the development of heart disease [18–20]. For instance, MC4R has been shown to independently regulate glucose homeostasis, and mutations in this gene may contribute to diabetes and cardiovascular disease (CVD) [21–24]. Previously, 30-week-old mice lacking the *Mc4r* gene were shown to produce increased levels of reactive oxygen species (ROS), which can have toxic effects on cardiomyocytes, resulting in reduced myocardial contractility and increased left ventricular diameter (LVIDd), leading to dilated cardiomyopathy and heart failure (HF) [18]. Puder *et al.* [25] reported that the *Mc4r* gene significantly reduced left ventricular mass (LV mass)/body surface area (BSA) and left ventricular end-diastolic volume/BSA, which in turn affected the normal function of the heart. In addition, the brain leptin‒MC4R pathway controls the involvement of perivascular sympathetic system in cardiovascular disease development [26,27]. Activation of the brain leptin-MC4R pathway has also been recently demonstrated to normalize and protect cardiac function after myocardial infarction and heart failure, suggesting that *Mc4r* may be a potential target for cardiac therapy [28]. Although most studies have confirmed the role of *Mc4r* in causing advanced heart disease, the mechanism of early intervention of this gene in heart disease is unclear and requires further elucidation.

This study aims to investigate the role of *Mc4r* in affecting myocardial remodeling and cardiac function in early heart disease. Utilizing the *Mc4r* knockout mice, we studied the effects of Mc*4r* deficiency on cardiac function and gene expression in 6–12 weeks old mice, and combined these data with bioinformatics analysis to screen for signaling pathways and key hub genes that are enriched mainly in differentially expressed genes (DEGs). Furthermore, we predicted the targets of the hub genes and the effective molecules targeting them. This study aims to elucidate the molecular alteration caused by *Mc4r* deficiency before the onset of heart disease symptoms, to provide theoretical guidance for subsequent prevention and early intervention, given that numerous disease mechanisms have already been studied.

## 2. Results

### 2.1. Effect of *Mc4r* knockout on cardiac function in mice

**Fig 1:**
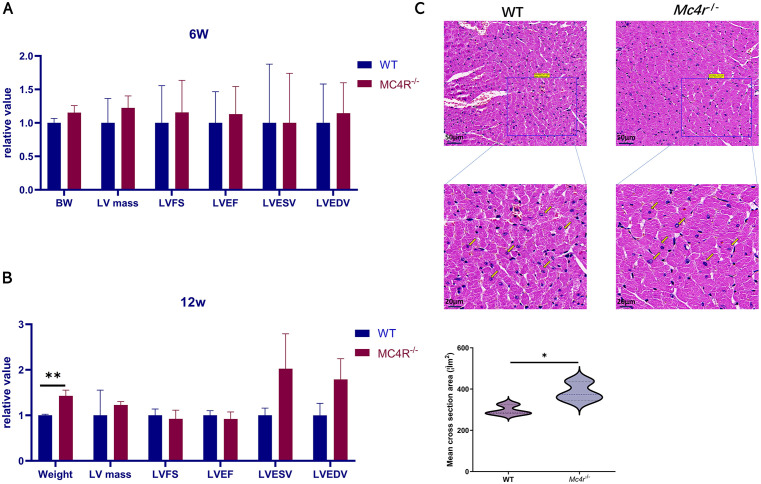
Analysis of cardiac function indicators and left ventricular myocardial tissue in Mc4r knockout mice and wild-type mice. **(A)** Comparison plots of body weight (BW) left ventricular mass (LV mass), LV fractional shortening (LVFS), LV ejection fraction (LVEF), LV end-systolic volume (LVESV) and LV end-diastolic volume (LVEDV) between *Mc4r* KO and WT mice at 6 weeks of age. **(B)** Comparison plots of body weight (BW) left ventricular mass (LV mass), LV fractional shortening (LVFS), LV ejection fraction (LVEF), LV end-systolic volume (LVESV) and LV end-diastolic volume (LVEDV) between Mc4r KO and WT mice at 12 weeks of age (multiple unpaired t-test, **p = 0.0043) **(C)** HE staining of left ventricular myocardial tissue in 12-week-old Mc4r KO mice and WT mice (×20, × 60) and comparison of average cross-sectional area of myocardial cells in Mc4r KO mice and WT mice. Yellow arrows refer to WT normal cardiomyocytes and Mc4r KO cardiomyocytes with increased cross-sectional area (unpaired t-test, *p = 0.045). WT (wild type), *p < 0.05, **p < 0.01.

To analyze the effect of the *Mc4r* gene on early cardiac function in mice, we used echocardiographic assays to compare the cardiac function of the *Mc4r*^*-*^KO mice and the WT mice at 6 and 12 weeks (**Fig 1A and 1B)**. At 6 and 12 weeks, no significant differences in the LV mass, the LV shortening fraction (LVFS), the LV ejection fraction (LVEF), the LV end-systolic volume (LVESV) or the LV end-diastolic volume (LVEDV) were detected between the *Mc4r*^*-*^KO and WT mice. At 12 weeks, the body weight of the *Mc4r* KO mice was significantly greater than that of the control mice (**Fig 1B)**. These studies suggest that *Mc4r*^*-/-*^ does not affect the measured cardiac parameters early in cardiac development. However, the abnormal increase in body weight of 12-week-old *Mc4r*^*-/-*^ mice suggests that *Mc4r* deficiency has caused adverse effects on mouse hearts. The decrease in LV mass/BSA and left ventricular end-diastolic volume/BSA in 12-week-old mice is also in line with previous findings by Puder *et al* [25].

### 2.2. Effects of *Mc4r* knockout on morphology, fibrosis and apoptosis in mice cardiomyocytes

To further confirm the effect of *Mc4r* deletion on mouse heart and explore its mechanism, we performed Hematoxylin-Eosin Staining (HE), Masson and terminal deoxynucleotidyl-transferase-mediated dUTP nick end-labeling (TUNEL) staining of the left ventricle in the hearts of the *Mc4r* KO mice and the WT mice at 12weeks. HE staining revealed that the mean cross-sectional area of cardiomyocytes was significantly greater in the *Mc4r*^*-*^KO mice than in the WT mice (**Fig 1C**). The cardiomyocytes were red in color under Masson staining, the cells were neatly arranged and clearly structured, and the collagen fibers were blue in color. Compared with those from the WT mice, cardiomyocytes from the *Mc4r*^*-*^KO mice did not show significant collagen fiber deposition (**S1A Fig**). TUNEL staining revealed no significant difference in the proportion of positive cells in the *Mc4r* KO mice compared with the WT mice (**S1B Fig**), indicating that *Mc4r* has no significant effect on apoptosis in early-stage mice. Combined with the abnormal weight gain of mice at 12 weeks of age without significant changes in cardiac function indexes, we speculate that *Mc4r* deficiency has some effects on mouse heart cells at this time, but it is still in a compensatory stage. The above results suggest that cardiac disease caused by *Mc4r* knockdown may lead to an increase in the cross-sectional area of cardiomyocytes in the early stage, but has little effect on the degree of fibrosis and apoptosis of cardiomyocytes.

### 2.3. Differentially expressed gene enrichment analysis

**Fig 2:**
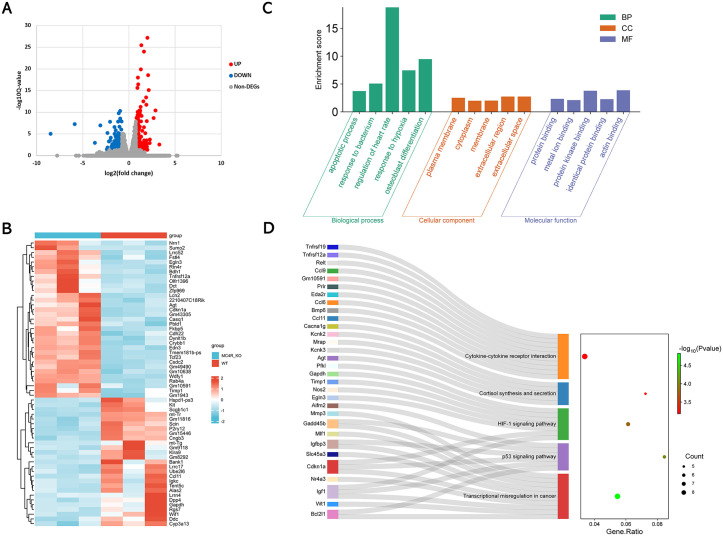
Differential gene expression and enrichment analysis in Mc4r KO mouse and WT mouse cardiomyocytes. **(A)** Volcano plot displaying differential gene expression, where red represents up-regulated genes and blue represents down-regulated genes; **(B)** heatmap displaying DEGs (|fold change (FC)| > 1.5, Q < 0.05); **(C)** GO enrichment analysis of DEGs, the top five GO term (biological process, cellular component, molecular function) branches with significant enrichment were presented; **(D)** KEGG enrichment analysis of DEGs. Significant enrichment was found in five signaling pathways, and P values have been shown in the Fig. KEGG: Kyoto Encyclopedia of Genes and Genomes; GO: Gene Ontology; DEGs: differentially expressed genes.

In order to further study the mechanism of *Mc4r* in the early stage of heart disease, lock the signaling pathways and find the targets for subsequent treatment, we first performed enrichment analysis of differentially expressed genes. Through RNA sequencing analysis, we screened 381 differentially expressed genes. Compared with those in the WT mice, there were 207 significantly upregulated DEGs and 174 significantly down-regulated DEGs in the *Mc4r*^*-*^KO mice. These DEGs could be involved in subsequent functional enrichment analysis, protein-protein interaction (PPI) network construction, and subsequent screening of early diagnostic markers. In Fig 2A **and** 2B, we used a volcano map and a heatmap to show the expression levels of DEGs between the two groups. **Fig 2C** and **2D** show the results of GO: Gene Ontology (GO) and Kyoto Encyclopedia of Genes and Genomes (KEGG) enrichment analyses. We selected the top five terms in the GO enrichment analysis, of which the biological processes (BP) that were significantly enriched were the apoptotic process, response to bacterium, regulation of heart rate, response to hypoxia and osteoblast differentiation; the significantly enriched cellular component (CC) terms were the plasma membrane, cytoplasm, membrane, extracellular region, and extracellular space; and the significantly enriched molecular function (MF) terms were protein binding, metal ion binding, protein kinase binding, identical protein binding and actin binding. DEGs were significantly enriched in five KEGG pathways: transcriptional misregulation in cancer, the p53 signaling pathway, the HIF-1 signaling pathway, cortisol synthesis and secretion and cytokine‒cytokine receptor interaction.This result reveals the main function of the selected DEGs, and provides a basis for further research on the mechanism construction of *Mc4r* in early heart disease and the development of drug targets.

### 2.4. Screening and analysis of hub genes

**Fig 3:**
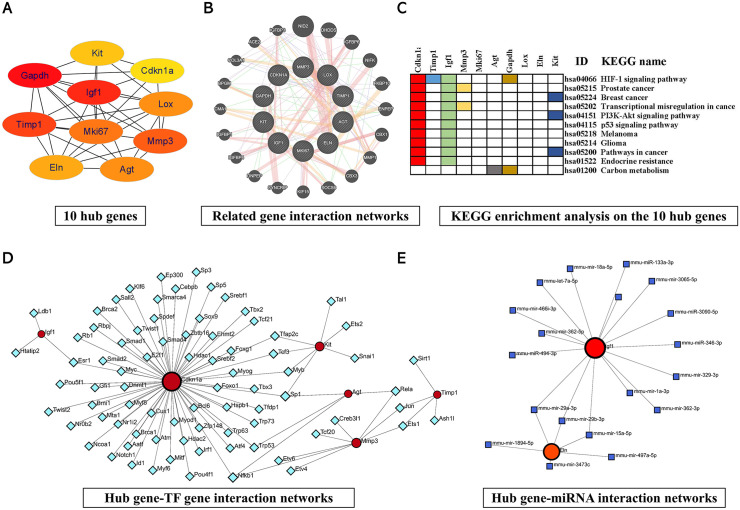
Protein‒protein interaction network of differentially expressed genes and modular analysis. **(A)** Hub gene regulatory network of the top 10 genes. (Only the selected key genes were retained) **(B)** MMP3, LOX, TIMP1, AGT, ELN, MK167, IGF1, KIT, GAPDH, and CDKN1A and their related gene interaction networks. Different colored lines represent different sources of evidence. **(C)** Signaling pathway enrichment of the top 10 hub genes. The presence of a color block indicates that the gene corresponding to the column of the color block is enriched in the signal pathway corresponding to the row. **(D)** Interaction analysis of hub genes and TF-genes. Red indicates Hub genes; Other colors indicate TF-genes. **(E)** Interaction analysis of 10 hub genes and miRNAs. Red indicates Hub genes; Other colors indicate miRNAs.

We constructed a PPI network for the DEGs via the STRING website. The generated network includes 342 nodes and 409 edges (**S2 Fig**). To filter the core cluster, we imported the generated PPI network into Cytoscape software and used the MCODE plug-in for cluster analysis. With the CytoHubba plug-in, we selected 10 hub genes according to their score values from high to low*: glyceraldehyde‐3‐phosphate dehydrogenase (Gapdh), insulin-like growth factor 1 (Igf1), tissue inhibitor matrix metalloproteinase 1 (Timp1), matrixmetalloproteinase 3 (Mmp3), Mki67, angiotensinogen (Agt), lysyl oxidase (Lox), elastin (Eln), KIT Proto-Oncogene (Kit), and cyclin-dependent kinase-inhibitor 1 (Cdkn1a)* (**Fig 3A)**. Using the GeneMANIA online website tool, we identified complex interactions between 10 hub genes and 20 other genes, such as *nidogen-2 (Nid2), dehydrodolichyl diphosphate synthase (Dhdds), and IGF-binding protein 6 (Igfbp6),* including physical interactions, predicted interactions, coexpression, colocalization, and genetic interactions (**Fig 3B)**. In addition, we performed KEGG enrichment analysis on the 10 hub genes. The pathways and gene enrichment of the significantly enriched genes are shown in **Fig 3C**. Transcription factors and miRNAs are the main “switches” in the cellular regulatory network, and hub genes are often the intersection of regulatory effects.We predicted and visualized the interactions of the TF genes and miRNAs with the 10 hub genes (**Fig 3D and 3E)**. The results shed light on the hierarchical control logic in the regulatory network, which helps us to identify the key targets and reveal the regulatory mechanism of these hub genes in heart disease, especially in the early stage of heart disease.

### 2.5. Hub gene expression verification and consistency analysis

**Fig 4:**
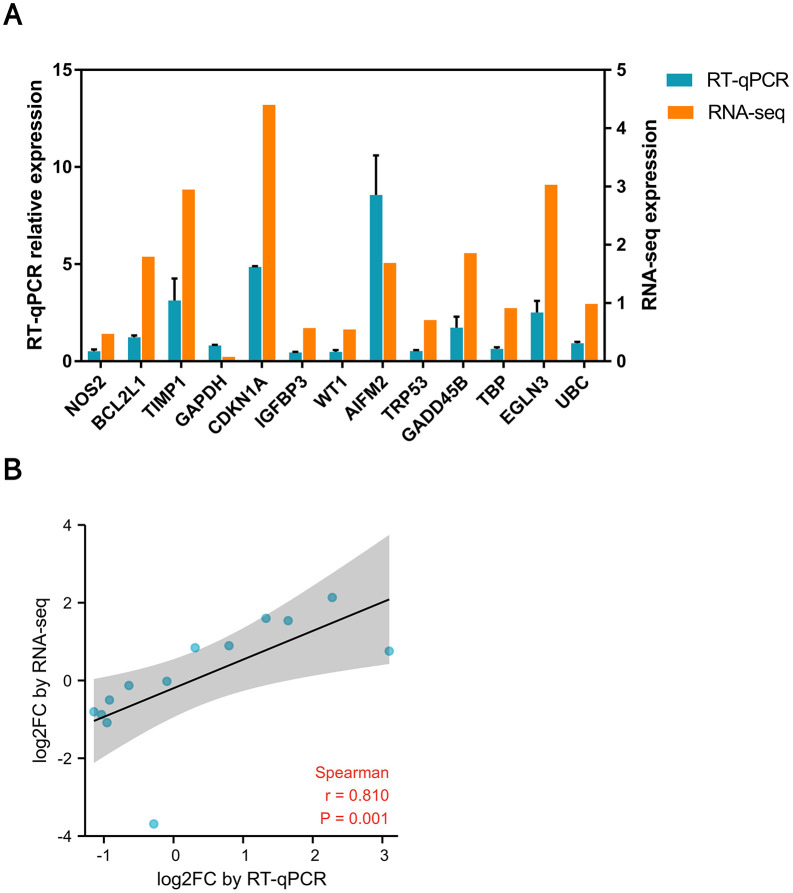
Quantitative validation of hub gene expression and scatter plot analysis. **(A)** Quantitative validation results of the hub genes. Gene expression levels were normalized to the control group. RT-qPCR values were calculated using the 2^(-ΔΔCt) method. RNA-seq values are presented as TPM fold change. **(B)** The consistency analysis of the scatter plot between the quantitative results and sequencing results showed that r > 0.8 indicated that the quantitative results were consistent with the RNA-seq results.

We selected 13 genes for RT‒qPCR validation and conducted a consistency analysis of the quantitative validation results through scatter trend plots. The results revealed that the expression trends of the quantitative genes were highly consistent with the RNA-seq results **(Fig 4**). This also further validates the reliability of our sequencing results.

### 2.6. Interaction between the hub genes and drugs

**Fig 5:**
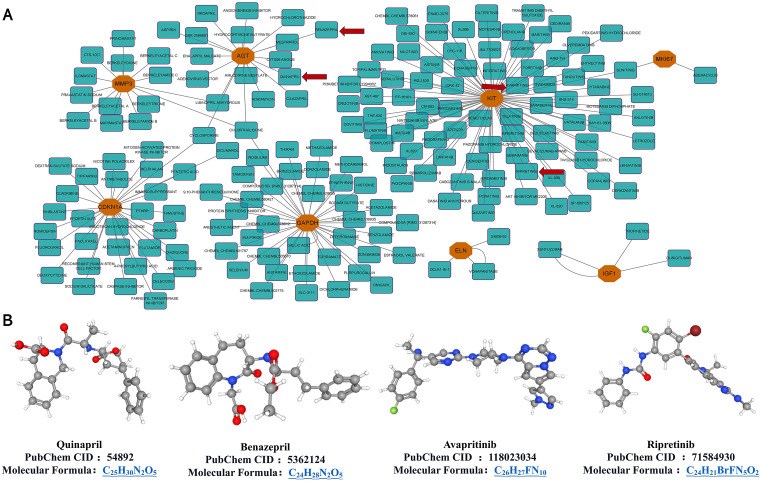
Hub gene interactions with drugs. **(A)** Prediction results of potential small-molecule drugs on the basis of 10 hub genes. Orange hexagons represent Hub genes, blue boxes represent drugs, and red arrows refer to drugs with an interaction score > 1.5 and regulatory approval. **(B)** Three-dimensional structures of the four potential small-molecule drugs screened out.

We obtained multiple drug‒gene interaction pairs via DGIDB analysis and screened the potential drug targets of each hub gene. The results revealed that eight hub genes, *Cdkn1a, Eln, Igf1, Agt, Gapdh, Kit, Mki67*, and *Mmp3*, potentially interact with drugs **(Fig 5A**). Afterward, we obtained four drugs based on screening criteria with interaction scores>1.5 approved by regulatory authorities: quinapril, benazepril, avapritinib and ripretinib **(Fig 5B**).

**Fig 6:**
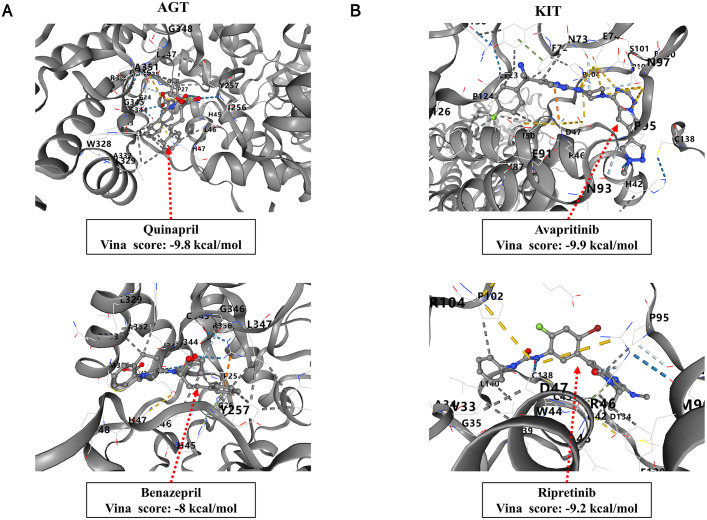
The results of molecular docking. **(A)** Docking of the 3D structure of AGT with two compounds to evaluate its binding affinity as a potential therapeutic target. **(B)** Docking of the 3D structure of KIT with two compounds to evaluate its binding affinity as a potential therapeutic target.

Two drugs target two molecules, Agt and Kit. Afterward, we explored the possible therapeutic mechanisms of these drugs through molecular docking simulations. We identified the 3D structures of the hub genes Agt and Kit and docked them with compounds to evaluate their binding affinity as potential therapeutic targets. The structure and binding energy between proteins and molecules are shown in **Fig 6**. It is generally believed that when the Vina score is < −7.0 kcal/mol, the binding activity between the two is strong. Among them, the binding energy of avapritinib is relatively high.

## 3. Methods

### 3.1. Mouse handling

*Mc4r* KO mice were purchased from Beijing Weitongda Biotechnology Co., Ltd. All the mouse lines were maintained on a C57B/6/N background and reproduced via the het-het mating strategy to generate homozygous knockout (*Mc4r*^*−/−*^) and wild-type (WT) littermate controls. The number of animals in each group is 3. When conducting RNA analysis, the age of the mice was 12 weeks. Under specific pathogen-free (SPF) conditions, the mice were subjected to a 12-hour light/dark cycle and housed at 25 °C. During this time, the mice had free access to water and standard chow. Mice of the indicated ages were subjected to echocardiography. For all necropsy studies, the mice were deeply anesthetized with 5 mg/kg tribromoethanol and then sacrificed by decapitation, followed by immediate collection of heart tissue. The resulting samples were collected in precooled phosphate-buffered saline to wash away residual blood. All the tissue samples were stored in liquid nitrogen for immediate freezing and then transferred to a −80 °C refrigerator for long-term storage until further analysis.

### 3.2. Echocardiography

Echocardiography was performed via the VEVOÒ 2100 digital ultrasound system (Visual Sonics; Toronto, Ontario). Studies were performed using an MS400 18–38 MHz transducer. Mice were anesthetized with 1.5% isoflurane and maintained on a heated platform in the supine position with continuous oxygen supply. Two-dimensional (2D) and M-mode images were acquired from the parasternal long-axis view. LV parameters, including LV mass, LVFS, LVEF, LVESV, and LVEDV, were measured using the built-in software VisualSonics Software v2.2. The measurements were made in a blinded manner via the LV trace function.

### 3.3. RNA isolation and cDNA preparation

RNA was isolated via the TransZol Up Kit (TransGenBiotech) according to the manufacturer’s instructions. After the samples were centrifuged, the uppermost mixture containing RNA was removed, isopropanol was added at a ratio of 1:1, the mixture was mixed well, and the mixture was centrifuged again after standing at room temperature for 10 minutes. Then, 75% ethanol was added for centrifugation, and the mixture was washed twice. After brief drying, 20 μL of RNase-free water was added to resuspend the RNA pellet, and the Optical Density (OD) value was assessed to determine the RNA concentration. The RNA was used as a template for reverse transcription and cDNA synthesis via TransScript IV One-Step gDNA Removal and cDNA Synthesis SuperMix (TransGen Biotech). Finally, the cDNA obtained via reverse transcription was stored at −20 °C for future use.

### 3.4. RT‒qPCR

Each component (premixed, upstream and downstream primers, template, Rox and sterile water) was added to the reaction mixture in a certain proportion on ice. Specific primers and TB Green™ Premix Ex Taq™ II (TaKaRa) were used for RT‒PCR. The PCR protocol was as follows: 40 cycles with 95 °C for 30 s in a PCR instrument; 95 °C, 5 s; 57 °C, 30 s; and 72 °C, 30 s per cycle. The dissolution curve was run at 60 °C, and the temperature was increased by 0.5 °C every 5 s until the cycle stopped at 90 °C. UBC and TBP were used as internal controls. The primers used are shown in Table 1.

**Table 1:** Sequences for gene primers.

Gene Symbol	RT Forward Primer(5’-3’)	RT Reverse Primer(5’-3’)	Accession Number	Product Length (bp)
NOS2	GTTCTCAGCCCAACAATACAAGA	GTGGACGGGTCGATGTCAC	NM_010927.4	127
BCL2L1	GACAAGGAGATGCAGGTATTGG	TCCCGTAGAGATCCACAAAAGT	NM_001289716.2	124
TIMP1	GCAACTCGGACCTGGTCATAA	CGGCCCGTGATGAGAAACT	NM_001044384.1	226
GAPDH	TTGTCTCCTGCGACTTCAACA	ACCAGGAAATGAGCTTGACAAAG	NM_001289726.2	99
CDKN1A	CCTGGTGATGTCCGACCTG	CCATGAGCGCATCGCAATC	NM_007669.5	103
IGFBP3	CCAGGAAACATCAGTGAGTCC	GGATGGAACTTGGAATCGGTCA	NM_008343.2	101
WT1	CAAGGACTGCGAGAGAAGGTTT	TGGTGTGGGTCTTCAGATGGT	NM_144783.2	137
AIFM2	CTGCCTACCGCAGTGCATT	ACGCCATCATTTCTGCCCA	NM_178058.4	265
TRP53	CTCTCCCCCGCAAAAGAAAAA	CGGAACATCTCGAAGCGTTTA	NM_011640.3	84
GADD45B	GAGGCGGCCAAACTGATGAAT	CGCAGCAGAACGACTGGAT	NM_008655.1	127
TBP	CCTTGTACCCTTCACCAATGAC	ACAGCCAAGATTCACGGTAGA	NM_013684.3	119
EGLN3	AGGCAATGGTGGCTTGCTATC	GCGTCCCAATTCTTATTCAGGT	NM_028133.2	118
UBC	CCAGTGTTACCACCAAGAAG	ACCCAAGAACAAGCACAAGG	NM_019639.4	94

### 3.5. Hematoxylin‒eosin staining

Heart tissues were fixed in 4% paraformaldehyde for 24 hours, dehydrated through a graded ethanol series, embedded in paraffin, and sectioned at 5 μm thickness. The slices were soaked twice with xylene or absolute ethanol and then rinsed with tap water after immersion in 75% alcohol for 5 minutes. Then, the sections were stained with hematoxylin staining solution, differentiated with differentiation solution, and returned to blue with blue returning solution. After the above three steps, all the samples were rinsed with running water. Slices were dehydrated with 85% and 95% alcohol, stained with eosin, soaked in absolute ethanol and xylene several times, and sealed with transparent neutral gum. Finally, the images were collected and analyzed via microscopy.

### 3.6. Masson’s trichrome staining

The tissue sample sections were placed in xylene and soaked twice for dewaxing, each time for 20 min. The tissue samples were subsequently soaked in absolute ethanol two times for 5 min each and then soaked in 75% ethanol for 5 min for hydration. The tissue samples were chromized with potassium dichromate overnight, and then, the sections were washed with tap water. The mixture was incubated with the prepared hematoxylin for 1 min, and the excess dye was removed with tap water after dyeing. Then, 1% hydrochloric acid ethanol was used for differentiation for 30 s, and the tissue sample was rinsed with tap water. Then, the tissue samples were fully stained with Ponceau acid fuchsin solution for 6 min, and the floating color was removed by washing with water. The samples were treated with a phosphomolybdic acid aqueous solution for 1 min. Without washing with water, the cells were counterstained with aniline blue solution for 2–30 s and differentiated with 1% glacial acetic acid for 1 min. After staining, the tissue sections were eluted with alcohol twice with an absolute ethanol gradient. After dehydration, the samples were soaked in anhydrous ethanol and xylene for 5 minutes each for transparency. After the samples were sealed with neutral gum, the film was examined under a microscope.

### 3.7. TUNEL assay

The tissue samples were dewaxed by soaking in xylene 3 times for 10 minutes each, hydrated by soaking in absolute ethanol 3 times, and washed with distilled water. In the room temperature environment, circles were drawn around the tissue with a histochemical pen, proteinase K and Phosphate Buffered Saline (PBS) were added to proteinase K working solution at a ratio of 1:9, the mixture was added to the circle, the tissue samples were treated for 22 minutes, the tissue samples were soaked in PBS, decolorized, and washed three times on a shaking table for 5 minutes each. The sample sections were gently dried, 0.1% Triton was added to each well, the samples were incubated at room temperature for 20 minutes, and the samples were washed three times with PBS on a shaker. The sections were gently dried and incubated with buffer at room temperature for 10 min. The TDT enzyme, dUTP and buffer were mixed at a ratio of 1:5:50 to prepare the TUNEL detection solution, which was dropped into the circle and incubated in a 37 °C incubator with a wet box in the dark for 2 hours. Then, the nuclei were counterstained with DAPI staining solution, incubated in the dark for 10 min at room temperature, fully decolorized, washed three times with PBS, and then sealed with an antifluorescence quenching sealing agent. After the samples were sealed, they were observed and photographed under a microscope.

### 3.8. RNA sequencing

Total RNA was extracted from tissues or cells using standard extraction methods, followed by strict quality assessment of RNA samples primarily, through precise detection of RNA integrity using the Agilent 2100 Bioanalyzer. PolyA-tailed mRNA was enriched using Oligo(dT) magnetic beads, and the isolated mRNA was randomly fragmented in NEB Fragmentation Buffer with divalent cations, following the standard NEB library construction protocol. Following library construction, preliminary quantification was conducted using the Qubit 2.0 Fluorometer, libraries were diluted to 1.5 ng/ μL, and the insert size of libraries was detected using the Agilent 2100 Bioanalyzer. Once the insert size was confirmed to meet expectations, qRT-PCR was used to accurately quantify the effective library concentration (library effective concentration > 2 nM) to ensure library quality. Qualified libraries were pooled according to effective concentration and desired sequencing data volume for Illumina sequencing. To ensure data analysis quality and reliability, raw data underwent a filtering process, which included the removal of adapter-containing reads, reads with N (N indicates undetermined base information), and low-quality reads (reads where >50% of bases had Qphred≤20 over the entire read length). Concurrently, Q20, Q30, and GC content were calculated for clean data. All subsequent analyses were conducted using high-quality analyses based on clean data. After raw data filtering, sequencing error rate checking, and GC content distribution evaluation, clean reads for subsequent analysis were obtained (Numerical data are available in **S1 Table**). The reference genome index was constructed using HISAT2 (v2.0.5), and paired-end clean reads were aligned to the reference genome using HISAT2 (v2.0.5). featureCounts (v1.5.0-p3) was employed to calculate reads mapped to each gene(Numerical data are available in **S2 Table**). FPKM for each gene was then calculated based on gene length and the number of mapped reads. For samples that included biological replicates, DESeq2 (v1.20.0) was ultilized for differential expression analysis between two comparison groups. The Benjamini and Hochberg method was applied to adjust obtained p-values (padj) in order to control the false discovery rate. The above sequencing was commissioned to Beijing Novogene Bioinformatics Technology Co., Ltd.

### 3.9. Identification of DEGs

We set log |fold change (FC)| > 0.75 and Q < 0.05 to identify DEGs between the control group and the experimental group. The sequencing results revealed 381 DEGs, among which, compared with those in the control group, 207 DEGs were significantly upregulated, and 174 DEGs were significantly downregulated. We generated a volcano map and a heatmap to show the expression of differentially expressed genes between the control group and the experimental group. The volcano map was drawn via Excel software, and the heatmap was visualized via R software (R (4.2.1) version, R package: ComplexHetmap [2.13.1]).

### 3.10. GO and KEGG enrichment analysis

We used KOBAS (KEGG Orthology Based Annotation System) online software to analyze the DEGs for GO and KEGG enrichment. The version used was KOBAS 3.0 (http://bioinfo.org/kobas/). We used gene list enrichment in the enrichment module, which is based on the over-representation analysis method. A corrected P value <0.05 was considered to indicate significant enrichment [29]. GO and KEGG data were plotted at https://www.bioinformatics.com.cn, an online platform for data analysis and visualization [30].

### 3.11. Network construction and hub gene analysis

We used the STRING online website to construct a PPI network for the DEGs (https://cn.string-db.org/). After that, we used the MCODE plug-in in Cytoscape software to analyze the module and set the parameters as follows: node score cutoff = 0.2; k-core = 2; and max. depth = 100. We selected the most important cluster for follow-up analysis and applied the CytoHubba plug-in to screen the cluster for hub genes. The screening method identified the top 10 nodes ranked by maximal clique centrality (MCC). Protein interaction analysis of the hub genes was performed via the GeneMANIA online website tool, and KEGG analysis of the hub genes was performed via KOBAS online software [31,32]. The obtained hub genes were uploaded to NetworkAnalyst (version 3.0, https://www.networkanalyst.ca/) to construct TF gene interaction networks and miRNA interaction networks [33].

### 3.12. Drug target analysis

Potentially active drugs were analyzed via DGIDB software (https://dgidb.org/), and quinapril, benazepril, avapritinib, and ripretinib were obtained through screening (approved by regulatory authorities, interaction score>1.5) [34]. Molecular docking simulation technology was used to explore the possible therapeutic mechanisms of these drugs. The small molecule structure file of the drug was identified via the PubChem website (3D structure image of CID 54892 (quinapril), PubChem Identifier: CID 54892, URL: https://pubchem.ncbi.nlm.nih.gov/compound/54892#section=3D-Conformer. 3D structure image of CID 5362124 (benazepril), PubChem Identifier: CID 5362124, URL: https://pubchem.ncbi.nlm.nih.gov/compound/5362124#section=3D-Conformer. 3D structure image of CID 118023034 (avapritinib), PubChem Identifier: CID 118023034, URL: https://pubchem.ncbi.nlm.nih.gov/compound/118023034#section=3D-Conformer. 3D structure image of CID 71584930 (ripretinib), PubChem Identifier: CID 71584930, URL: https://pubchem.ncbi.nlm.nih.gov/compound/71584930#section=3D-Conformer) [35]. The protein structure of the molecule was determined via the UniProtKB website (https://www.uniprot.org/uniprotkb/) [36], and then, the docking between the molecule and the drug was scored via the CB-DOCK2 website (https://cadd.labshare.cn/cb-dock2/index.php) [37]. Structure-based blind docking in CB Dock2 was selected, and AutoDock Vina-based docking was performed in each detected cavity. The potential binding sites of the queried ligands were ranked on the basis of the Vina score (kcal/mol), and the top-ranked sites were selected.

### 3.13. Statistical analysis

The DEGs in the sequencing data were corrected via the Eseq2 method, and a Q value <0.05 was considered to indicate a significant difference. All data from the experiments were statistically analyzed via GraphPad Prism software. Statistical significance was determined by t tests or one-way ANOVA. p < 0.05 was considered to indicate a significant difference. RT-qPCR and RNA-seq data.are available in S3 Table, the data generated in this study are available in S4 Table.

### 3.14. Ethics approval

The management of all mouse lines was approved by the Animal Ethical and Welfare Committee of Jining Medical University (Approval number: JNMC-2022-DW-003) during the entire experimental procedure. We confirm that all experiments were performed in accordance with relevant guidelines and regulations. We confirm that this study was reported in accordance with the ARRIVE guidelines.

## 4. Discussion

Litt *et al.‘s* study suggests that *Mc4r* deficiency leads to progressive cardiomyopathy in an age-dependent manner [18]. Reduced cardiac dilation and contractility were observed in 26-week-old male Mc4r knockout mice, as well as in female mice. The fractional shortening, myocardial contractility, and ejection fraction (EF) of the mouse heart in this age group were significantly reduced, while the LV diameter was significantly increased. Gava FN *et al.* found that infusing Mc4r agonists into the heart after myocardial infarction in rats significantly increased EF and FS, maintained the contractile ability of cardiomyocytes, thereby reducing adverse cardiac remodeling and protecting cardiac function [28]. Sex hormones influence the structure and physiological function of the heart, and some studies have shown differences in wall thickness, LV ejection fraction, and ECG of the heart after puberty, and that males have a higher risk of cardiovascular disease than females [38]. In this regard, we took into account that females subjects may experience interference with experimental variables by fluctuations in the estrogen cycle, an occurrence not present in males. Based on this point of view, many studies in the literature also use male mice for relevant investigations [39–41].

In accordance with established stages of cardiac development in mouse models, we selected 6-week-old and 12-week-old mice as experimental subjects. At 6 weeks of age, cardiomyocyte proliferation ceases, and subsequent cardiac growth occurs primarily through hypertrophy (cell volume enlargement). By 12 weeks, the mouse heart reaches full maturity and achieves fully developed function [42]. Our cardiac function measurements on 6–12-week-old *Mc4r* knockout male mice showed that compared to WT mice, the indicators of cardiac function we tested LV mass, LVFS, LVEF, LVESV, and LVEDV were not changed. However, in the cardiomyocytes of *Mc4r* knockout mice at 12 weeks, the cross-sectional area is increased. More importantly, mRNA sequencing results from mouse left ventricular tissue showed that *Mc4r* deficiency mice cardiomyocytes underwent changes at various molecular levels, which may precede the development of cardiac malfunction at late age. Based on the sequencing results, we screened hub genes and the enriched signaling pathways. The functional exploration of hub genes reveals a significant association between them and cardiac function. We speculate that the hub molecules detected in the hearts of young *Mc4r* deficiency mice may become promising candidates for early prevention of related cardiac disease. We acknowledge that exploratory analysis at the 6- and 12-week age points were limited by a small sample size (n = 3 per group), which reduces the statistical power of detect subtle changes. However, the phenotypes observed across three independent experimental cohorts exhibited high reproducibility, suggesting that the biological effects were robust enough to be detectable despite the limited sample size. Furthermore, while early molecular data only provided a basis for future validation to a certain extent, we plan to further investigate the core mechanisms of cardiac dysfunction by expanding the sample size.

The cardiomyocyte cross-sectional area is the key to assessing cardiac hypertrophy, myocardial hypertrophy may lead to heart disease, such as heart failure, which is one of the most important risk factors for cardiovascular death [43–46]. We therefore hypothesize that the enlarged cardiomyocyte cross-sectional area caused by *Mc4r* KO may be an earlier pathological change in heart disease. In addition, cardiomyocyte apoptosis and fibrosis can be observed in many cardiovascular diseases. Myocardial apoptosis is an important form of cell death in the early stages of myocardial infarction and can promote heart remodeling and further progression to heart failure [47,48]. Cardiomyocyte fibrosis can lead to heart failure, malignant arrhythmias, sudden cardiac death and other serious conditions. This process is often observed in the advanced stages of various heart diseases [49,50]. Our Masson and TUNEL results revealed that *Mc4r* deletion did not affect cardiomyocyte fibrosis or apoptosis, suggesting that the mice may be in the early stage of heart disease. In summary, we showed that the knockout of *Mc4r* could alter the cross-sectional area of cardiomyocytes but did not affect cardiomyocyte apoptosis or fibrosis.

We further explored the effect of *Mc4r* deficiency on cardiomyocytes at the molecular level via transcriptome sequencing. By GO and KEGG enrichment analyses, we identified that HIF-1 pathway and P53 pathway were alterated in Mc4r deficiency mice heart.The HIF-1 signaling have been shown to participated in a variety of diseases, including arterial dissection, pulmonary hypertension, atherosclerosis, and myocardial infarction [51,52]. High expression of HIF-1 inhibits the production of *Serca2* and reduces the contractility of the heart, which in turn affects the normal function of the heart and leads to the development of heart disease [53]. However, it has also been shown that the HIF-1 signaling pathway can play a role in protecting cardiac function by regulating the expression of miRNAs. Under hypoxic or low-oxygen conditions, the HIF-1 pathway can reduce apoptosis and injury in cardiomyocytes by inhibiting the expression of miR-20b-5p and miR-135b [54–56]. This pathway also promotes angiogenesis by regulating miR-126 expression during myocardial ischemia [57]. These contradictory results may be due to differences in downstream genes regulated by HIF-1. In addition, there is a close association between HIF-1α signaling and cardiomyocyte morphology. Gao *et al.* [58] demonstrated that when this signaling pathway is activated, it reduces the energy metabolism of the rat myocardium and increases the cross-sectional area of cardiac myocytes. This finding is similar to the results we obtained from HE staining of the left ventricular myocardium.

In our study, the DEGs in heart samples from the WT mice and *Mc4r* KO mice were enriched in the P53 signaling pathway. The P53 signaling pathway plays an important role in tumor suppression. Studies in heart-related diseases have revealed that P53 signaling has a role in maintaining homeostasis in cardiac tissue and is considered to be a master regulator of the cardiac transcriptome [59]. P53 has been reported to play an important role as a microRNA target gene in mediating cardiac hypertrophy and cardiomyocyte morphological remodeling [60]. Zhan *et al.* [61] reported that miR-128 down-regulation reduced the extent of increase in cardiomyocyte cross-sectional area through targeted inhibition of p53, thereby alleviating the effects of chronic angiotensin activity on myocardial hypertrophy. We speculate that P53 inhibition may be an early compensatory outcome of cardiac disease, impeding the progression of heart disease to a malignant degree. However, these inferences require functional validation, such as analysis of protein expression by Western blot or hypoxia response assays, as our current data only show pathway enrichment and lack direct evidence of protein activity or cellular function. In addition, P53 is involved in myocarditis of a diabetic nature due to elevated blood glucose. Inhibition of the P53 pathway not only slows cardiomyocyte senescence but also promotes the expression of hypoxia-inducible factor-1a in the body to maintain normal cardiac function [62]. Thus, a decrease in P53 during the compensatory phase may play a role in stress protection. When activated, the P53 pathway promotes acetylation of the P53 gene and the expression of dynamin-related protein, which is involved in the apoptosis of cardiomyocytes, leading to heart failure, among other events [63,64]. However, our apoptotic results revealed no statistically significant difference in cardiomyocyte apoptosis in the *Mc4r*^*-*^KO mouse P53 signaling pathway compared with that in the controls, possibly because apoptosis occurs at a later stage of heart disease.

Through the construction of a PPI network and the analysis of the hub genes, we identified 10 hub genes, among which five key hub genes (*Cdkn1*, *Igf1*, *Gadph, Agt* and *Kit*) are closely associated with the regulation of cardiac function and targeted therapy for heart-related diseases. CDKN1, also known as P21, is involved in the regulation of the cell cycle as a cyclin-dependent kinase inhibitor [65]. Our results revealed that *Cdkn1* gene expression was significantly upregulated after *Mc4r* knockdown, which may be associated with the onset of early compensation for cardiac disease. Notably, the upregulation of Cdkn1a (a P53 target gene) was consistent with an increase in cardiomyocyte cross-sectional area (Fig 1C), suggesting a potential link between P53 pathway activation and myocardial remodeling. This gene plays an important role in increasing LV systolic and diastolic rates, limiting excessive increases in LV end-systolic and diastolic diameters, and repairing damaged cardiomyocytes. These effects enhance basal cardiac function and reduce the damage caused to the heart during ischemia and reperfusion [66,67]. Additionally, *Cdkn1a* is highly expressed specifically in certain hypertrophic cardiomyocytes [68]. These results are all consistent with our experimental results that *Cdkn1* gene up-regulation is involved in the occurrence of cardiac disease. In mouse cardiomyocytes, a lack of *Cdkn1a* (*P21Cip*) hinders the proliferation of cardiomyocytes and eventually leads to heart failure [69]. This finding is inconsistent with our results, possibly because of the early compensation of Cdkn1 or differential regulation of the *Mc4r* gene in this study. The above findings suggest that *Mc4r* knockdown may lead to myocardial hypertrophy and thus affect the normal physiological function of the heart by upregulating *Cdkn1c* and thereby regulating the morphology of cardiomyocytes; however, the specific molecular mechanisms involved need to be further investigated.

IGF-1 may act as a cardiomyocyte growth factor involved in cardiovascular diseases such as heart failure, atherosclerosis, and the cardioprotective effects of cardiovascular aging [70]. It has been demonstrated the therapeutic potential of *Igf-1* in cardiac diseases. Davis *et al.* [71] used biotinylated nanofibers for targeted delivery of *Igf-1* to cardiomyocytes to induce phosphorylation activation of the downstream mediator *Akt*. This process not only increased the cross-sectional area of cardiomyocytes but also alleviated cardiac contractile disorders after myocardial infarction. Other studies have also shown that the activating IGF-1 signaling restores cardiac dysfunction by increasing the left ventricular EF in isoprenaline-infused mice and mice with diabetic cardiomyopathy [72,73]. GADPH is an important reactive enzyme in human tissue glycolysis that reduces the probability of heart disease mainly by inhibiting apoptosis. For example, activation or increased expression of *Gapdh* in cardiomyocytes facilitates the protection of cardiomyocytes from apoptosis and thus improves cardiac function [74]. Moreover, Sukhanov *et al.* [75] reported that enhanced expression of *Gapdh* in vascular smooth muscle cells under redox-sensitive mechanisms protected against _H2O2_-induced apoptosis. *Gapdh* can also overcatalyze the partial conversion of cardiomyocyte glycolysis intermediates to lipid synthesis products during ischemia to maximize the protection of cardiac function [76]. In the present study, *Igf-1* and *Gapdh* gene expression was significantly down-regulated after *Mc4r* knockdown, which is consistent with the above findings, suggesting that *Mc4r* knockdown may affect cardiac function by down-regulating *Igf-1* and *Gapdh*.

In addition, we screened potential drug targets for heart-related diseases caused by the *Mc4r* gene through DGIDB analysis and further searched for potential drugs that may target these molecules, obtaining four drugs: quinapril, benazepril, avapritinib and ripretinib. These four drugs target two molecules, *Agt* and *Kit*. Kit encodes a type 3 receptor tyrosine kinase [77], and Amini H *et al.* reported that cardiac progenitor cells can promote the regeneration and repair of heart tissue after injury by expressing c-kit [78]. Furthermore, Bryan *et al.* found in Tie2^CreERT2^ transgenic mice, deletion of the essential cardiogenic transcription factor Gata4 led to defective tube formation and a reduction in CD31 + hypoendothelial cells, resulting in severe cardiac endothelial cell expansion and differentiation defects [79]. The vast majority of mutations in Kit are sensitive to avapritinib and ripretinib, and ripretinib, a Kit inhibitor, has been applied in the treatment of advanced gastrointestinal stromal tumors [80]. Additionally, Avapritinib, a potent and selective oral inhibitor, suppresses proliferation and reduces viability in pancreatic cancer cells, and exhibits strong efficacy in gastrointestinal stromal tumors (GISTs) [81]. These findings indicate that Kit may improve cardiac function by participating in the repair process of myocardial tissue. Therefore, avapritinib and ripretinib may be potential drugs for the treatment of heart-related diseases using targeting Kit. Several cardiovascular diseases, such as myocardial infarction and coronary heart disease, have been linked to AGT polymorphisms [82]. In addition, the Met174 allele of AGT has been shown to cause abnormal expression of AGT plasma levels, leading to accelerated vasoconstriction and lipid accumulation [83]. We speculate that the p.Thr174Met polymorphism of AGT affects the expression of AGT, accelerates vascular constriction, slows blood circulation, and results in insufficient blood supply to the heart. Moreover, Rong *et al.* found that hepatocyte-specific AGT knockout in a mouse model of sepsis-induced cardiac dysfunction was associated with significant improvement in cardiac function significantly improved cardiac function and prolonged survival time [84]. These studies suggest that AGT plays an important role in the pathogenesis of cardiac disease. Quinapril and benazepril have been identified as agents that modulate gene expression within the AGT pathway [85]. For example, quinapril potentiates a beneficial role in the prevention of restenosis after percutaneous coronary intervention (PCI) in patients with AGT T purists [86]. These findings are similar to our research findings. These two drugs may help patients return to a normal blood flow rate by targeting AGT, thereby treating heart disease.

In summary, we suggest that in the young stage of mice, Mc4r deficiency may affect the microstructure of the heart through cardiomyocytes enlargement, but does not affect FS, LV weight etc. In young *Mc4r* deficient mice, although there were no organic lesions in the heart, abnormal changes had already occurred at the molecular level which may serve as early diagnostic indicators. We hypothesize that the regulatory mechanism of early cardiac changes in *Mc4r* deficient mice may involve the p53 signaling pathway and HIF-1 signaling pathway. Based on the relevant hub genes, we screened potential drugs and molecular targets for treatment of heart related diseases caused by the Mc4r gene deficiency. This study emphasized the HIF-1/P53 molecular mechanism in the *Mc4r* gene in regulating the early onset of heart disease, providing new concepts and theoretical guidance for subsequent early diagnosis and preventive treatment strategies.

## 5. Limitations and future directions

While our study reveals early molecular alterations in MC4R-deficient hearts, several limitations should be acknowledged. First, the small sample size (n = 3 per group) and preclinical mouse model may restrict extrapolation to human pathophysiology. To address these gaps, future work should validate findings in larger cohorts, including clinical samples from obese individuals with MC4R mutations. Second, although we identified p53/HIF-1 pathways as key mechanisms, further exploration of oxidative stress and metabolic reprogramming could provide a more comprehensive understanding of MC4R-related cardiac remodeling. Finally, protein-level validation (e.g., Western blot) and therapeutic testing of Agt/Kit-targeting drugs (e.g., avapritinib) in disease models are needed to translate our discoveries into clinical applications. Our data establish a foundation for these future investigations.

## Supporting information

S1 Fig Left ventricular myocardial tissue was analyzed by Masson staining and TUNEL staining. (A) Results of Masson staining (10x, 40x) of the left ventricular myocardial tissue of Mc4r KO mice versus WT mice and comparison of the percentage area of collagen fibers in the myocardial cells of Mc4r KO mice versus WT mice. *p < 0.05 (B) TUNEL staining and TUNEL-positive cell rate of left ventricular myocardial tissue in Mc4r-KO mice and WT mice.
(TIF)

S2 Fig PPI network of DEGs established via the STRING online website. (TIF)

S1 Table Clean reads for subsequent analysis. (PDF)

S2 Table Reads mapped to each gene. (PDF)

S3 Table RT-qPCR and RNA-seq data. (PDF)

S4 Table The data generated in this study. (PDF)

## Abbreviations

MC4R: Melanocortin-4 receptor
CVD: Cardiovascular disease
HF: Heart failure
DEGs: Differentially expressed genes
LV mass: Left ventricular mass
BSA: body surface area
LVIDd: left ventricular diameter
EF: ejection fraction
AGT: angiotensinogen
FS: fractional shortening
