# Supplementary figures and images for: Molecular remodeling of the myocardium in mice with melanocortin-4 receptor deletion before cardiac function impairment
Source: PLoS One. 2026 Jan 30;21(1):e0340465. doi: 10.1371/journal.pone.0340465 (PMC12857938; doi:10.1371/journal.pone.0340465)

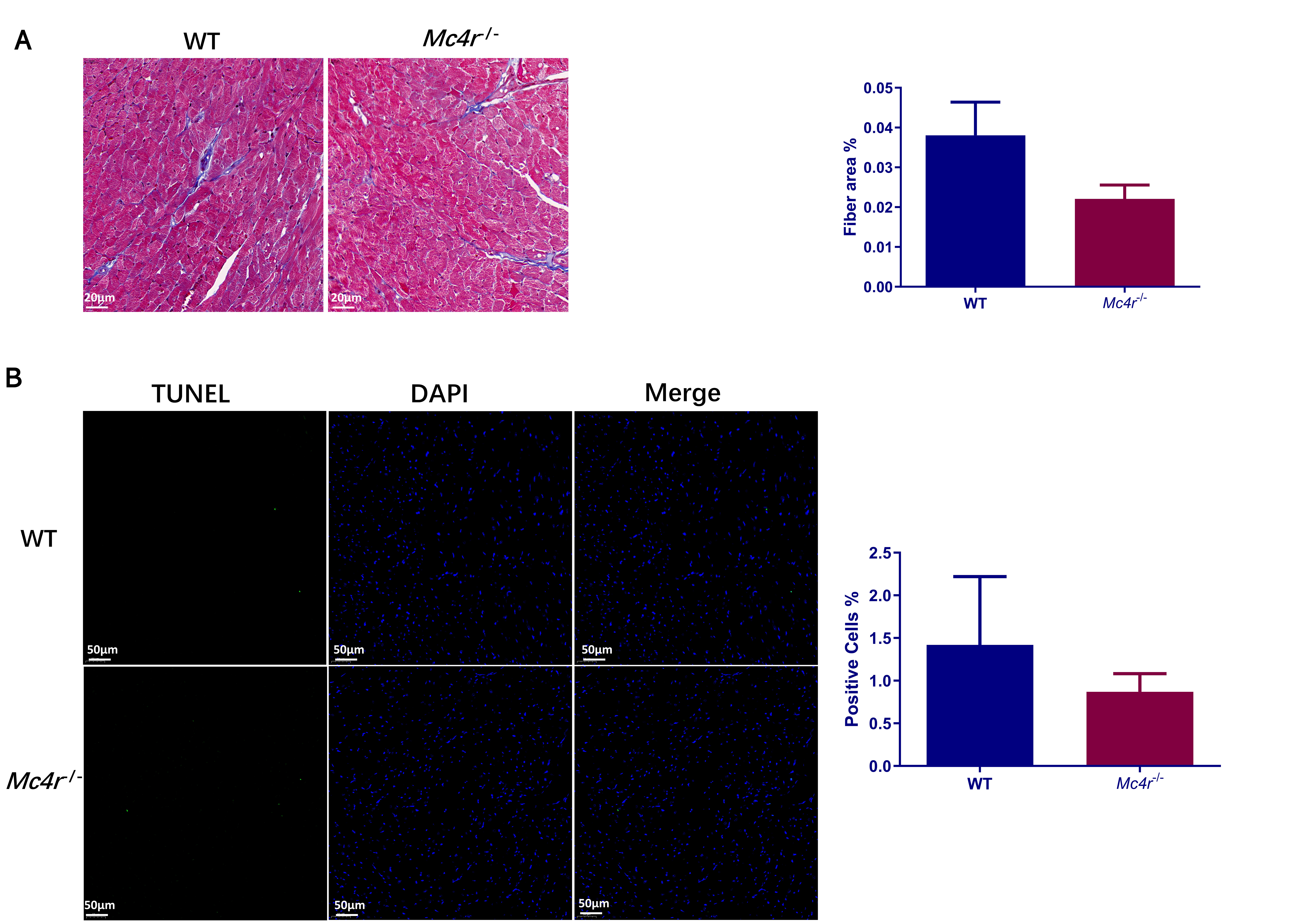

Supplement: S1 Fig — (A) Results of Masson staining (10x, 40x) of the left ventricular myocardial tissue of Mc4r KO mice versus WT mice and comparison of the percentage area of collagen fibers in the myocardial cells of Mc4r KO mice versus WT mice. *p < 0.05 (B) TUNEL staining and TUNEL-positive cell rate of left ventricular myocardial tissue in Mc4r-KO mice and WT mice. (TIF) [file pone.0340465.s001.tif]

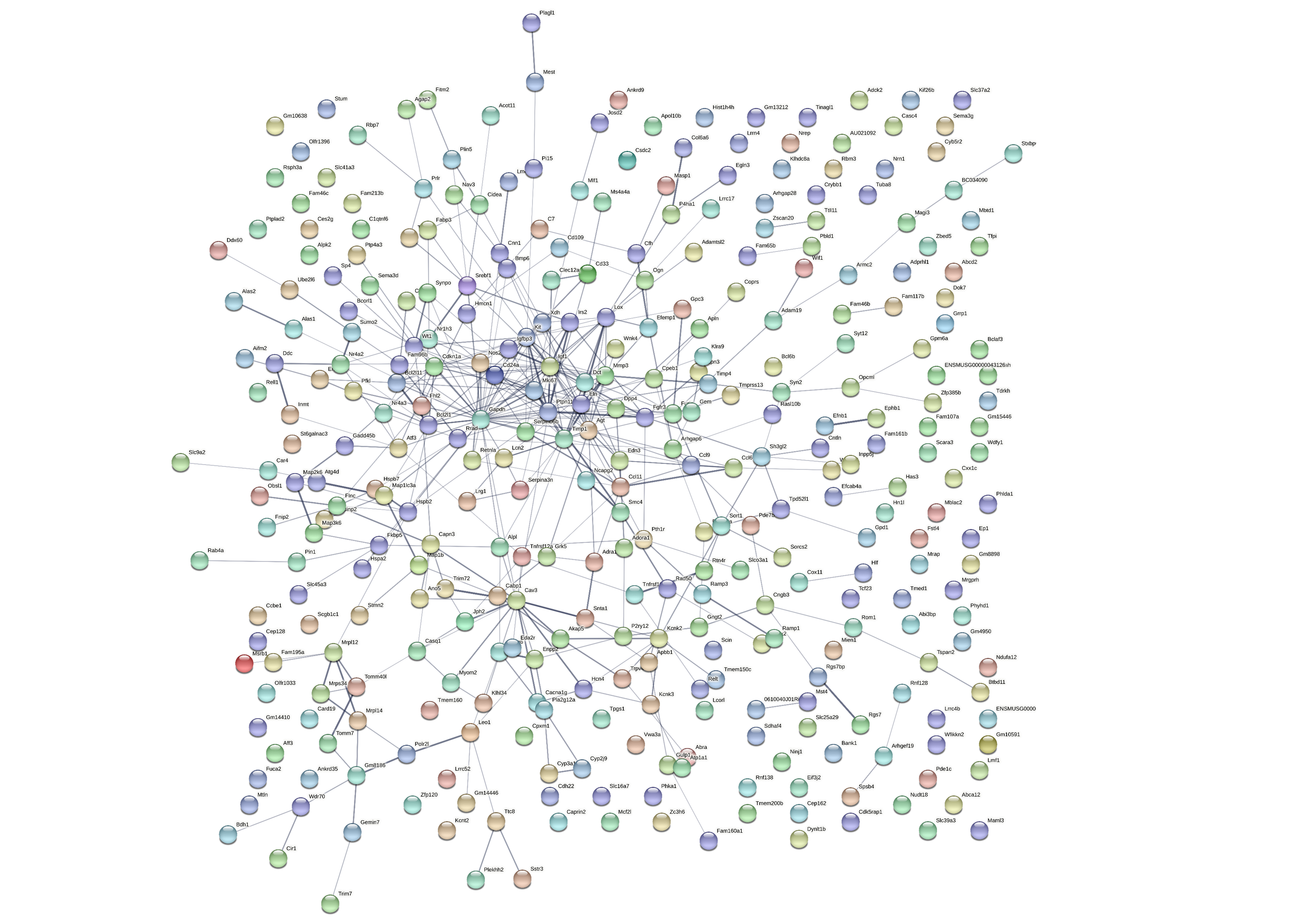

Supplement: S2 Fig — (TIF) [file pone.0340465.s002.tif]
