# Supplementary material for: Molecular remodeling of the myocardium in mice with melanocortin-4 receptor deletion before cardiac function impairment
Source: PLoS One. 2026 Jan 30;21(1):e0340465. doi: 10.1371/journal.pone.0340465 (PMC12857938; doi:10.1371/journal.pone.0340465)
Supplement: S1 Table — (PDF) [file pone.0340465.s003.pdf]

Exported data									
sample	library	raw_reads	raw_bases	clean_reads	clean_bases	error_rate	Q20	Q30	GC_pct
WT_1	FRAS210178909-1r	45121188	6.77G	43441994	6.52G	0.03	97.79	93.5	44.54
WT_2	FRAS210178910-1r	45476222	6.82G	42837698	6.43G	0.03	97.94	93.94	44.95
WT_3	FRAS210178911-1r	46285984	6.94G	44877486	6.73G	0.03	97.75	93.59	46.92
MC4R_KO_1	FRAS210178912-1r	42154486	6.32G	40485668	6.07G	0.03	97.63	93.32	47.31
MC4R_KO_2	FRAS210178913-1r	44523106	6.68G	42993338	6.45G	0.03	97.75	93.54	46.95
MC4R_KO_3	FRAS210178914-1r	44839148	6.73G	43213024	6.48G	0.03	97.76	93.57	47.42
