# Supplementary material for: Molecular remodeling of the myocardium in mice with melanocortin-4 receptor deletion before cardiac function impairment
Source: PLoS One. 2026 Jan 30;21(1):e0340465. doi: 10.1371/journal.pone.0340465 (PMC12857938; doi:10.1371/journal.pone.0340465)
Supplement: S2 Table — (PDF) [file pone.0340465.s004.pdf]

Exported data											
sample	total_reads	total_map	unique_map	multi_map	read1_map	read2_map	positive_map	negative_map	splice_map	unsplice_map	proper_map
WT_1	43441994	42298151(97.37%)	33636861(77.43%)	8661290(19.94%)	16823599(38.73%)	16813262(38.7%)	16805293(38.68%)	16831568(38.74%)	11196765(25.77%)	22440096(51.66%)	32322438(74.4%)
WT_2	42837698	41656767(97.24%)	33679982(78.62%)	7976785(18.62%)	16833726(39.3%)	16846256(39.33%)	16822039(39.27%)	16857943(39.35%)	12156045(28.38%)	21523937(50.25%)	32153014(75.06%)
WT_3	44877486	43197862(96.26%)	36441926(81.2%)	6755936(15.05%)	18233356(40.63%)	18208570(40.57%)	18183343(40.52%)	18258583(40.69%)	14014435(31.23%)	22427491(49.97%)	35107650(78.23%)
MC4R_KO_1	40485668	39064793(96.49%)	33139344(81.85%)	5925449(14.64%)	16560375(40.9%)	16578969(40.95%)	16530829(40.83%)	16608515(41.02%)	13384417(33.06%)	19754927(48.79%)	31929198(78.87%)
MC4R_KO_2	42993338	41523452(96.58%)	34930197(81.25%)	6593255(15.34%)	17474762(40.65%)	17455435(40.6%)	17428696(40.54%)	17501501(40.71%)	13773195(32.04%)	21157002(49.21%)	33734150(78.46%)
MC4R_KO_3	43213024	41630455(96.34%)	35657329(82.52%)	5973126(13.82%)	17837840(41.28%)	17819489(41.24%)	17790446(41.17%)	17866883(41.35%)	14201673(32.86%)	21455656(49.65%)	34396946(79.6%)
