# Supplementary material for: Molecular remodeling of the myocardium in mice with melanocortin-4 receptor deletion before cardiac function impairment
Source: PLoS One. 2026 Jan 30;21(1):e0340465. doi: 10.1371/journal.pone.0340465 (PMC12857938; doi:10.1371/journal.pone.0340465)
Supplement: S3 Table — (PDF) [file pone.0340465.s005.pdf]

		RT-qPCR					RNA-seq			
					mean	log2FC by RT-qPCR	Group_2	Group_1		log2FC by RNA-seq
MC4R1	NOS2	0.430812	0.715169	0.401047	0.515676	-0.955462976	142.833635	303.4245	0.470739	-1.084959016
	BCL2L1	1.13942	1.417983	1.163056	1.240153	0.310518585	1133.95275	631.831	1.794709	0.84194814
	TIMP1	5.385251	2.218954	1.785534	3.129913	1.646122618	44.8214541	15.20294	2.948209	1.538034003
	GAPDH	0.87235	0.763485	0.829583	0.821806	-0.283129957	111.329208	1436.245	0.077514	-3.689403496
	CDKN1A	4.94128	4.815565	4.815884	4.857576	2.280236638	900.185558	204.5082	4.401709	2.134095519
	IGFBP3	0.471513	0.390168	0.496729	0.452803	-1.14304311	395.224478	690.3507	0.572498	-0.804455933
	WT1	0.578789	0.574351	0.310941	0.488027	-1.034967489	64.1950014	117.5294	0.546204	-0.877243778
	AIFM2	9.51652	11.52833	4.652469	8.565775	3.098583751	634.230826	375.3622	1.68965	0.755357903
	TRP53	0.5285	0.439234	0.617862	0.528532	-0.91993723	187.2023	264.9506	0.706555	-0.502069953
	GADD45B	1.192851	2.853854	1.165629	1.737444	0.796966804	144.91375	78.09794	1.855539	0.89340174
	TBP	0.672698	0.485303	0.766264	0.641421	-0.640655931	147.421824	161.4637	0.913034	-0.128635492
	EGLN3	3.202386	3.023847	1.307917	2.511383	1.328482237	1969.7277	650.2136	3.029355	1.5986701
	UBC	0.876115	1.061923	0.870585	0.936208	-0.095099438	1550.19307	1573.451	0.985219	-0.020898097
