# Supplementary material for: Molecular remodeling of the myocardium in mice with melanocortin-4 receptor deletion before cardiac function impairment
Source: PLoS One. 2026 Jan 30;21(1):e0340465. doi: 10.1371/journal.pone.0340465 (PMC12857938; doi:10.1371/journal.pone.0340465)
Supplement: S4 Table — (PDF) [file pone.0340465.s006.pdf]

Data for various indicators in 6-week-old mice

	Weight	LV mass	LVFS	LVEF	LVESV	LVEDV
WT	1.008929000	0.623565282	1.618950847	1.510145034	0.319025817	0.620520634
	1.062500000	1.349729946	0.835945495	0.895230402	0.690544322	0.711628359
	0.928571000	1.026704773	0.545103658	0.594624564	1.990429861	1.667851007
MC4R-/-	1.272321000	1.143745954	0.626597269	0.675828897	1.818056586	1.604674685
	1.071429000	1.429239427	1.271089798	1.245448871	0.805902299	1.134450676
	1.111607000	1.101173949	1.568028305	1.473266018	0.376041115	0.694550524

Data for various indicators in 12-week-old mice

	Weight	LV mass	LVFS	LVEF	LVESV	LVEDV
WT	0.975000000	0.586378299	0.874840138	0.908560371	0.818992776	0.723807915
	1.005000000	1.627696663	1.148093950	1.108240931	1.108847241	1.246354782
	1.020000000	0.785925038	0.977065912	0.983198698	1.072159983	1.029837302
MC4R-/-	1.406250000	1.244112504	0.858128382	0.877156986	1.649858654	1.410685132
	1.563750000	1.145995417	0.778369405	0.796418589	2.906334415	2.293306311
	1.316250000	1.290097422	1.137432366	1.092262801	1.514354518	1.665878512

	HE data
WT	326.1377000
	284.3478000
	281.6283000
Mc4r-/-	346.0827000
	438.0973000
	373.9192000

MASSON data

WT	0.029721
	0.029745
	0.054724
Mc4r-/-	0.028818
	0.017052
	0.020329

TUNEL data

WT	1.82
	0.5
	1.94
Mc4r-/-	0.79
	1.11
	0.71
